# Prevalence and Associated Factors of Complains on Depression, Anxiety, and Stress in University Students: An Extensive Population-Based Survey in China

**DOI:** 10.3389/fpsyg.2022.842378

**Published:** 2022-03-28

**Authors:** Yanling Yu, Wangwang Yan, Jiadan Yu, Yangfan Xu, Dan Wang, Yuling Wang

**Affiliations:** ^1^Rehabilitation Medicine Center, The Sixth Affiliated Hospital, Sun Yat-sen University, Guangzhou, China; ^2^Department of Sport Rehabilitation, Shanghai University of Sport, Shanghai, China; ^3^School of Medicine, Changsha Social Work College, Changsha, China

**Keywords:** depression, anxiety, stress, university students, epidemiological investigation

## Abstract

Mental health issues are becoming increasingly prevalent amongst university students. However, research on the psychological profile of the general university population is relatively limited. Thus, this study analyses the current state of university students’ psychological conditions; the demographic differences in depression, anxiety, and stress and the influencing factors. The objectives are to provide additional appropriate guidance in mental health for university students with different demographic characteristics. A cross-sectional study of 6,032 university students nationwide was conducted from October 2020 to January 2021. A randomized whole-group sampling method was used to select the study participants, and the 21-item Depression, Anxiety, and Stress Scale (DASS) was used. *P* < 0.05 in the final model were considered statistically significant. The number of university students with no complain of depression, anxiety, or stress was 3,751 (62.2%). The odds of developing complain of depression were higher amongst anxious respondents (AOR = 23.417, 95% CI: 19.706, 27.826) and senior year (AOR = 2.210, 95% CI: 1.657, 2.947) than their counterparts. Students with “myopia” were 1.263 times more likely to be anxious (AOR = 1.263, 95% CI: 1.042–1.530). In terms of “impaired” or not, impaired is defined as any injury, such as sprain, strain, and fracture, “impaired” university students were 1.321 times more likely to be anxious (AOR = 1.321, 95% CI: 1.064–1.641). Furthermore, history of impairment and myopia increased the odds of stress by 1.305 (AOR = 1.305, 95% CI: 1.022–1.667) and 1.305 (AOR = 1.305, 95% CI: 1.012–1.683), respectively. Myopia, physical-activity-related injury (PARI) and irrational eating habits are risk factors for complain of anxiety and stress. Males, upper grades, low parental education, and irrational eating habits are risk factors for complain of depression. Low physical activity levels are also an influential factor for complain of depression. DASS consists of interchangeable risk factors and multiple complains of DASS may coexist.

## Introduction

Depression, anxiety, and stress are significant indicators for mental health and the presence of these symptoms could have a negative effect on an individual if not identified early or addressed timely (Wang et al., 2020), The period of transition from late adolescence to adulthood (Arnett, 2000) is a developmentally challenging transition for most young people in this time in many countries. During this period, these people are in the midst of the senior year of their university education (Torres et al., 2017). University years are a critical period of development and the onset of most lifelong mental disorders occurs during this period (Kessler et al., 2007).

Mental health problems account for approximately one-sixth of the global burden of disease in adolescents (Mokdad et al., 2016). Approximately 10–20% of adolescents in the world have had mental health problems. In addition, these problems have become the main reasons for the subsequent development of psychological barriers, such as those leading to risk-taking behavior and autotomy, and even the occurrence of suicide (Wagner et al., 2017; Johnson and Heiderscheit, 2018). Numerous studies showed that people who attend a university have more severe mental health problems and a correspondingly higher prevalence of psychological disorders, particularly depression, anxiety, and stress, than those of the same age who do not attend university (Cvetkovski et al., 2012). Moreover, mental health issues are becoming more prevalent amongst university students. Nearly one-half of university students have moderate levels of stress, anxiety, and depression (Regehr et al., 2013). This case causes irreparable damage to families, the country, and society.

In the United States, nearly half of the university-age individuals suffer from a mental disturbance (Han et al., 2016). Similarly, over 20% of Chinese university students have had depression, and this proportion has grown in the old days (Sontag-Padilla et al., 2018; Liu et al., 2019a,b). China is now one of the fastest-growing economies in the world (AlJaber, 2020). Along with economic development, Chinese people’s living standards have increased significantly and their psychological state has changed extensively (Ye et al., 2020). In addition, China has changed many of its policies in recent years. For example, the Chinese government has abolished the one-child policy (Scharping, 2018) and simultaneously, China is still expanding its university advancement rate (Ye et al., 2020). All these changes not only reflect dramatic changes in the social environment but may also affect the psychological characteristics of Chinese university students. In addition, university students are a key force in determining a country’s economic growth and success and university years are a critical period from adolescence to adulthood (Cuijpers et al., 2019). Considering the changing social roles and declining social support, university students are more vulnerable to psychological conditions than before (Auerbach et al., 2018; Liu et al., 2019a,b). Therefore, reliable estimates of the epidemic rates of mental health problems in China’s changing environment are an important step in the effort to provide enhanced mental health care for university students.

In conclusion, the prevention and early treatment of mental health problems amongst university students is an important public health priority because of its effect on students’ lives, public health, society’s investment in university students, and the importance of university students to society’s future capital (Cuijpers et al., 2019). Psychological disorders could pose a serious threat to students’ academic achievement and affect their future career course of events at the same time. Therefore, identifying the factors that affect the mental health of university students is of great significance. On this basis, this study aimed to understand complain of anxiety, depression, stress and their related factors amongst Chinese university students.

## Materials and Methods

### Design

According to the principle of stratified random whole-group sampling, four regions were divided in accordance with economic areas: Eastern, Central, Western, and North eastern regions. A cross-sectional study was conducted amongst Chinese university students on the prevalence of depression, anxiety, stress, and other related factors.

### Sample

Stratified random whole-group sampling method was used from October 2020 to January 2021, with the head of the selected school briefing the students on the purpose of the study.

The sample size was calculated based on pre-pandemic data. According to the results of similar surveys in other regions, the incidence of stress is about 34.5% (Ramón-Arbués et al., 2020). With a precision of 1.25% and a confidence interval of 95%, the recommended sample size was 5,556 using the sample size calculation tool. Taking into account a 10% questionnaire exclusion rate, the final sample size required was 6,173.

Simultaneously, the completion of the informed consent form was explained to the students to begin completing the electronic version of the questionnaire. Students were provided with the right to refuse to participate or withdraw from the study at no cost and their privacy and confidentiality were protected. A total of 6,714 questionnaires were returned and 6,032 students were included in this study after relevant screening and elimination.

The inclusion criteria were as follows: (1) university students enrolled in rehabilitation-related majors, including Freshman, Sophomore, Junior, Senior; (2) Those who signed the electronic informed consent form. Further, the exclusion criteria were as follows: (1) Non-rehabilitation related majors; (2) Suffered from severe cardiovascular disease; (3) Suffered from specific pathologies (e.g., epilepsy, amputation, etc.); (4) Inflammatory conditions; (5) A severe fracture; (6) Students with confused response logic; (7) Students with abnormal IPAQ long form data.

### Data Collection

The questionnaire included two main parts: social demographic characteristics and mental health. The socio demographic characteristics contained a series of alternating quantities commonly related to psychological puzzle amongst university students (Tao et al., 2002; Korhonen et al., 2017; Wahed and Hassan, 2017; Fernández et al., 2020; Lee and Feng, 2021; Solmi et al., 2021), that is, gender, grade, obesity, eating habits, family background, tobacco and alcohol consumption, physical activity, and diet. The variable body mass index (BMI) was calculated from self-reported weight and height (BMI = kg/m^2^). BMI is classified as follows: (1) low BMI (≤18.5 kg/m^2^), (2) normal BMI (18.5–23.9 kg/m^2^), (3) high BMI/obese (24.0–27.9 kg/m^2^), and (4) obese (≥28 kg/m^2^).

Physical activity was measured using the International Physical Activity Questionnaire (IPAQ) (Fogelholm et al., 2006). IPAQ is one of the widely used internationally validated questionnaires for measuring physical activity levels in adults (15–69 years). The physical activity counted in this questionnaire consists mainly of the type of activity (work, transportation, home gardening, leisure) and the intensity of the activity (walking and moderate and high intensity). This questionnaire is available in two versions (Macfarlane et al., 2011), long and short, and it has been used in studies of Chinese populations. Information on the dietary habits of university students were collected, including the frequency of intake of meat, fruits, vegetables, high-calorie drinks, tobacco, and alcohol.

Physical-activity-related injury is defined as “any injury, such as sprain, strain, and fracture, suffered in the process of participating in PAs or leisure time (Van Mechelen et al., 1992). A countable PARI event must have occurred within the past 12 months, with at least one of the following consequences: (1) having to stop the current PA (sport), (2) being unable or unable to fully participate in the next planned PA (sport), (3) being unable to attend class the next day, and (4) needing to seek medical support (Bloemers et al., 2012; Cai et al., 2018).

The psychological status assessment scale used in this survey is the simplified Chinese version of the 21-Item Depression, Anxiety, and Stress Scale (DASS-21). This scale, which was developed by Lovibond et al. in 1995, aims to distinguish and define common emotional disorders and help provide an auxiliary psychometric indicator for clinical diagnosis (Lovibond and Lovibond, 1995). DASS-21 is a streamlined edition of the DASS that has been revised to improve the efficiency of identifying and assessing symptoms of the corresponding mood disorders. Studies showed that the scale consists of three subscales, namely, depression, anxiety, and stress, containing seven questions each, for a total of 21 questions. The scale is rated on a three-point scale, 0 = not met, 1 = met or occasionally met, 2 = met or often met, and 3 = most or always. In DASS-D, 0–9 = no depression/normal, 10–13 = mild depression, 14–20 = moderate depression, 21–27 = severe depression, and >27 = very severe depression). In DASS-A, 0–7 = no anxiety/normal, 8–9 = mild anxiety, 10–14 = moderate anxiety, 15–19 = severe anxiety, and >19 = very severe anxiety. In DASS-S, 0–14 = no stress/normal, 15–18 = mild stress, 19–25 = moderate stress, 26–33 = severe stress, and >33 = very severe stress. DASS-21 is calculated by multiplying the sum of the subscale scores by two to obtain the final score for the three subscales. It has the same stable factor structure and equally good reliability as the full version of DASS, making this scale more suitable for use as a rapid screening tool in research and clinical settings (Antony et al., 1998; Crawford and Henry, 2003). The internal consistency of the three subscales ranged from 0.76 to 0.79 and that of the total scale reached 0.89 (Gong et al., 2010).

### Data Collection Procedures

This study was carried out in the form of electronic questionnaire survey. After the baseline survey was conducted in different regions and colleges across the country, the responsible persons of the eligible universities were communicated with and the questionnaires were distributed. All institutions were required to ask student participants to accomplish the questionnaires during the same time period. Before filling out the questionnaire, participants were asked to sign the informed consent form after reading the information sheet of the study and instructions for completing the survey.

### Data Collection Instrument and Quality Control

After a pre-survey of 40 interns was conducted at the Sixth Hospital of Sun Yat-sen University, the total scale Cronbach’s alpha coefficient was 0.816, which had good reliability.

### Data Analysis

The characteristics and dietary habits of the survey respondents were described by frequency and percentage. For the analysis of variability in psychological states of different subgroups, Chi-square analysis was used in the case of categorical variables, such as demographic characteristics: gender, place of birth, etc. Binary logistic regression analyses (reverse stepwise regression with an entry probability value of *p* = 0.05 and a removal value of *p* = 0.10) were also performed to identify the predictors of mental health (presence of anxiety, stress, and depression) in the sample. Data coding, processing, and analysis were completed using SPSS (version 25 for Windows, IBM Corp., Chicago, IL, United States), which accepts a significance level of *p* < 0.05.

### Ethical Considerations

Prior to the commencement, this study was reviewed and approved by the Ethics Committee of the Sixth Hospital of Sun Yat-sen University (IEC Ref: E2020035). This study complied with every national and international standard for ethical research in human subjects.

## Results

### Basic Characteristics

A total of 6,032 university students (67% female and 33% male) participated in this survey. The ages ranged from 15 to 25 years, with an average of 19.82 ± 1.429 years. The majority of students had a normal BMI (63.9%), the highest percentage was freshmen (38%) and the student population was rural (62.9%). In addition, 38.5% had a low level of physical activity and the incidence of injury was 13.1% (Table 1).

**TABLE 1:** Statistical table of basic characteristics of university students.

Variables		n (%)
Age	Mean ± SD	19.82 ± 1.429
Gender	Male	1,989 (33%)
	Female	4,043 (67%)
Grade	Freshman	2,294 (38%)
	Sophomore	1,617 (26.8%)
	Junior	1,522 (25.4%)
	Senior	5,88 (9.7%)
BMI	Mean ± SD	21.185 ± 3.8914
BMI grouping	BMI < 18.5 (low)	1,231 (20.4%)
	18.5 ≤ BMI ≤ 23.9 (normal)	3,853 (63.9%)
	24.0 ≤ BMI ≤ 27.9 (high)	696 (11.5%)
	BMI ≥ 28 (obese)	252 (4.2%)
Myopia	Yes	4,816 (79.8%)
	No	1,255 (20.2%)
Injury	Yes	792 (13.1%)
	No	5,240 (86.9%)
Mother’s educational level	Primary school and below	2,155 (35.1%)
	Junior high school or vocational high school	2,078 (34.4%)
	High school or junior college	1,017 (16.9%)
	Specialty	421 (7%)
	Bachelor’s degree and above	401 (6.6%)
Physical activity (MET* minutes/week)	Mean ± SD	2282.3 ± 2762.52
PA levels	Low	2,322 (38.5%)
	Moderate	1,957 (32.4%)
	High	1,753 (29.1%)
Father’s education level	Primary school and below	1,361 (22.6%)
	Junior high school or vocational high school	2,408 (39.9%)
	High school or junior college	1,236 (20.5%)
	Specialty	518 (8.6%)
	Bachelor’s degree and above	509 (8.4%)

*Metabolic equivalents (MET): the ratio of a person’s working metabolic rate relative to their resting metabolic rate.

### Dietary Habits

The eating habits of university students for the past week were surveyed and counted. Amongst these students, 1.7% did not consume meat in the last 7 days and 90.3% never smoked. The highest frequency of meat and fresh fruit intake was 2–3 days a week, with the least percentage of never consuming meat and fruit. Moreover, 35.8% of university students consumed fresh vegetables 4–5 days a week, with only 1.5% not consuming fresh vegetables. The highest percentage of university students hardly consumed carbonated drinks (37.2%) and more than half did not consume coffee in the last week. Then, the highest frequency of intake of high-calorie drinks, sugar and high-fat foods was the most frequent at 2–3 days. Furthermore, the percentages of university students who never smoked nor drank were 90.3 and 85.2%, respectively, and the percentages of those who smoked or drank often or always were 1.7 and 1.1%, respectively. Overall, the university students had good eating habits, with very few having poor eating habits (Table 2).

**TABLE 2:** Statistical table of university students’ eating habits.

Category	Never	Hardly ever	Sometimes	Often	Always
Meat	102(1.7%)	341(5.7%)	2,022(33.5%)	1,791(29.7%)	1,776(29.4%)
Fresh fruits	167(2.8%)	691(11.5%)	2,786(46.2%)	1,563(25.9%)	825(13.7%)
Fresh vegetables	90(1.5%)	283(4.7%)	1,936(32.1%)	2,159(35.8%)	1,564(25.9%)
Carbonated beverages	1,661(27.5%)	2,244(37.2%)	1,737(28.8%)	287(4.8%)	103(1.7%)
Coffee	3,409(56.5%)	1,445(24.0%)	890(14.8%)	186(3.1%)	102(1.7%)
High-calorie beverages	1,300(21.6%)	2,085(34.6%)	2,192(36.3%)	355(5.9%)	100(1.7%)
Sugar	723(12.0%)	1,329(22.0%)	2,764(45.8%)	850(14.1%)	366(6.1%)
High-fat foods	1,119(18.6%)	1,895(31.4%)	2,476(41.0%)	407(6.7%)	135(2.2%)
Cigarettes	5,445(90.3%)	226(3.7%)	261(4.3%)	41(0.7%)	59(1.0%)
Alcohol	5,139(85.2%)	542(9.0%)	285(4.7%)	37(0.6%)	29(0.5%)
					

### Statistical Results for Depression, Anxiety, and Stress Scale

Amongst the university students surveyed, 27.3 and 33.4% had mild to very severe complains of depression and anxiety, respectively. The levels of complain of depression and anxiety were higher in males than in females (*p* < 0.05). In addition, no significant difference was observed in complain of stress scores between males and females (12.7% of males and 11.6% of females). The scores of the depression, anxiety, and stress subscales of the university students surveyed were corresponding to the grading criteria provided by the scales. Moreover, the number of university students with different degrees of complain of depression accounted for 27.3% of the total number of students in this study. The number of students with different levels of complain of anxiety accounted for 33.4% of the total number of students. Amongst the different levels of complain of anxiety, moderate complain of anxiety accounted for the highest proportion (53.9%), whilst severe complain of anxiety accounted for the lowest (11.4%). The number of students with different levels of complain of stress accounted for 12%. Amongst the different levels of complain of stress, mild complain of stress accounted for the highest proportion (47.9%), whilst very severe complain of stress accounted for the lowest (5.8%).

**FIGURE 1:**
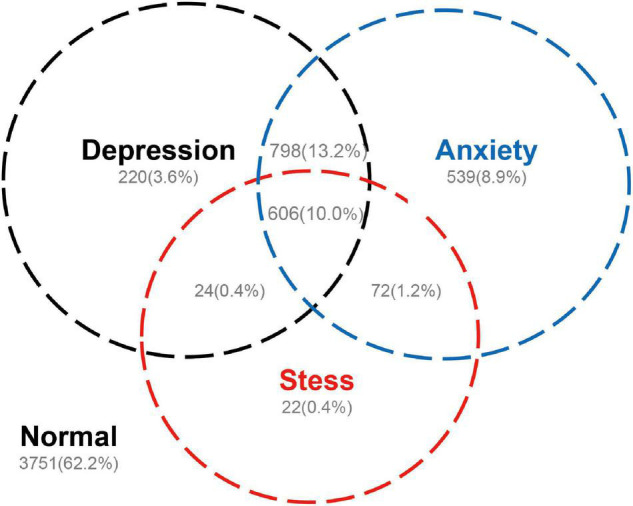
Coexistence of complains of depression, anxiety, and stress on the basis of the results from DASS-21 (*n* = 6,032).

Amongst the different levels of stress, the number of people without stress was the highest, followed by that without complain of depression, whilst the number of people without complain of anxiety was the lowest. The number of people with complain of anxiety was the highest in the mild level classification. The number of people with anxiety was the highest in the moderate, severe, and very-severe level classifications (Table 3). According to the results obtained from the DASS-21 questionnaire, 62.2% of the university students surveyed had none of the complains; 25.2% had two or more psychiatric complains and up to 10% experienced complains of anxiety, depression, and stress at the same time (Figure 1).

**TABLE 3:** Total and gender-based scores for DASS-21.

DASS-21a	Categories	Total (*n* = 6,032)	Men (*n* = 1,989)	Women (*n* = 4,043)	*P*
DASS-Db	No depression	4,384(72.7%)	1,391(69.9%)	2,993(74.0%)	<0.01
	Mild	620(10.3%)	177(8.9%)	443(11.0%)	
	Moderate	796(13.2%)	326(16.4%)	470(11.6%)	
	Severe	112(1.9%)	41(2.1%)	71(1.8%)	
	Extremely severe	120(2.0%)	54(2.7%)	66(1.6%)	
	Score Mean ± SD	5.83 ± 7.07	6.29 ± 7.64	5.61 ± 6.77	0.001
DASS-Ac	No anxiety	4,017(66.6%)	1,295(65.1%)	2,722(67.3%)	0.017
	Mild	442(7.3%)	128(6.4%)	314(7.8%)	
	Moderate	1,086(18.0%)	383(19.3%)	703(17.4%)	
	Severe	229(3.8%)	82(4.1%)	147(3.6%)	
	Extremely severe	258(4.3%)	101(5.1%)	157(3.9%)	
	Score Mean ± SD	6.07 ± 6.47	6.32 ± 7.12	5.94 ± 6.12	0.040
DASS-Sd	No stress	5,308(88.0%)	1,736(87.3%)	3,572(88.4%)	0.028
	Mild	347(5.8%)	114(5.7%)	233(5.8%)	
	Moderate	207(3.4%)	67(3.4%)	140(3.5%)	
	Severe	128(2.1%)	49(2.5%)	79(2.0%)	
	Extremely severe	42(0.7%)	23(1.2%)	19(0.5%)	
	Score Mean ± SD	7.03 ± 7.42	7.23 ± 7.94	6.93 ± 7.15	NS*

* NS, Not significant; DASS-21a, 21-item DASS-21 Depression, Anxiety and Stress Scale; DASS-Db, seven-item DASS-21 Depression Subscale; DASS-Ac, seven-item DASS-21 Anxiety Subscale; DASS-Sd, seven-item DASS-21 Stress Subscale.

The final binary logistic regression model showed that being male, being in the fourth year, having a father with a low level of education, never eating fresh vegetables, always drinking carbonated drinks and always consuming sugar were risk factors for complain of depression. Then, myopia, impairment and always consuming high-fat foods were influential factors for complain of anxiety and stress. Furthermore, complain of anxiety, depression and stress were influential factors for one another (*p* < 0.05, Tables 4–6).

**TABLE 4:** Factors related to complains of depression of university students.

Factors	Independent variables	DASS-D		*p*	AOR (95% CI)
		Yes	No		
Gender	Male	598	1,391		1
	Female	1,050	2,993	0.006	0.781 (0.655∼0.931)
Grade	Freshman	540	1,754	< 0.001	1
	Sophomore	456	1,161	< 0.001	1.458 (1.185∼1.795)
	Junior	463	1,070	< 0.001	1.706 (1.384∼2.104)
	Senior	189	399	< 0.001	2.210 (1.657∼2.947)
Father’s education level	Primary school and below	430	931	0.01	1
	Junior high school or vocational high school	638	1,770	0.003	0.728 (0.590∼0.899)
	High school or junior college	317	919	0.002	0.676 (0.528∼0.866)
	Specialty	146	372	0.059	0.734 (0.532∼1.012)
	Bachelor’s degree and above	117	392	0.016	0.656 (0.466∼0.923)
Carbonated drinks	Never	358	1,303	0.003	1
	Hardly ever	558	1,686	0.871	1.018 (0.820∼1.264)
	Sometimes	568	1,169	0.002	1.432 (1.140∼1.798)
	Often	121	166	0.144	1.348 (0.903∼2.014)
	Always	43	60	0.077	1.772 (0.940∼3.338)
Sugar	Never	135	588	0.049	1
	Hardly ever	341	988	0.007	1.561 (1.132∼2.152)
	Sometimes	806	1,958	0.005	1.529 (1.137∼2.057)
	Often	255	595	0.013	1.557 (1.100∼2.205)
	Always	111	255	0.02	1.671 (1.083∼2.577)
Stress	No	1,018	4,290		1
	Yes	630	94	< 0.001	7.147 (5.487∼9.311)
Anxiety	No	244	3,773		1
	Yes	1,404	611	< 0.001	23.417 (19.706∼27.826)
	Constants			< 0.001	0.047

*DASS-D, Depression Anxiety Stress Scalees-Dpression; AOR, Adjusted odds ratio; CI, Confidence interval.*

**TABLE 5:** Factors related to complain of anxiety of university students.

Factors	Independent variables	DASS-A		*p*	AOR (95% CI)
		Yes	No		
Myopia	Yes	1,642	3,174		1
	No	373	843	0.017	1.263 (1.042∼1.530)
Injury	No	1,695	3,545		1
	Yes	320	472	0.012	1.321 (1.064∼1.641)
High fat category	Never	279	840	< 0.001	1
	Hardly ever	569	1,326	0.048	1.262 (1.002∼1.589)
	Sometimes	927	1,549	< 0.001	1.556 (1.251∼1.937)
	Often	174	233	0.001	1.807 (1.285∼2.541)
	Always	66	69	0.012	1.993 (1.165∼3.408)
Stress	No	1,337	3,971		1
	Yes	678	46	< 0.001	1.824 (1.080∼18.114)
Depression	No	611	3,773		1
	Yes	1,404	244	< 0.001	1.537 (1.032∼26.687)
	Constants			< 0.001	0.074

*DASS-A, Depression Anxiety Stress Scalees-Anxiety; AOR, Adjusted odds ratio; CI, Confidence interval.*

**TABLE 6:** Factors related to complains of stress of university students.

Factors	Independent variables	DASS-S		*p*	AOR (95% CI)
		Yes	No		
Myopia	Yes	601	4,215		1
	No	123	1,093	0.033	1.305 (1.022∼1.667)
Injury	No	595	4,645		1
	Yes	129	663	0.04	1.305 (1.012∼1.683)
High fat category	Never	102	1,017	0.011	1
	Hardly ever	187	1,708	0.541	1.901 (1.645∼1.259)
	Sometimes	316	2,160	0.73	1.944 (1.683∼1.307)
	Often	79	328	0.155	1.372 (1.887∼2.121)
	Always	40	95	0.011	1.285 (1.211∼4.312)
Anxiety	No	46	3,971		1
	Yes	678	1,337	< 0.001	1.842 (1.125∼18.074)
Depression	No	94	4,290		1
	Yes	630	1,018	< 0.001	1.432 (1.720∼9.656)
	Constants			< 0.001	0.008

*DASS-A, Depression Anxiety Stress Scalees-Stress; AOR, Adjusted odds ratio; CI, Confidence interval.*

### Factors Associated With Depression Amongst University Students

After univariate analysis was conducted, factors that had a significant effect on depression were selected for multifactorial analysis. The results showed that gender, grade, father’s education, carbonated drinks, sugar intake, complain of anxiety, and stress were significantly associated with complain of depression. Females were less likely to be depressed than males (AOR = 0.781; 95% CI: 0.655–0.931). The odds of being depressed were 2.21 times higher in the fourth year than in the first year (AOR = 2.21, 95% CI: 1.657–2.947). The prevalence of complain of depression was higher amongst those whose fathers had a bachelor’s degree or higher education than those whose fathers only finished primary school or below (AOR = 0.656, 95% CI: 0.466–0.923). The university students who always consumed carbonated drinks and sugars were higher than those who never did (AOR = 1.772, 95% CI: 0.940–3.338; AOR = 1.671, 95% CI: 1.083–2.577, Table 4).

### Factors Associated With Anxiety Amongst University Students

After univariate analysis was conducted, factors that had a significant effect on anxiety were selected for multifactor analysis. The results showed that myopia, the presence of injury, high fatty-food intake, complain of depression, and stress were significantly associated with complain of anxiety. The odds of having anxiety were higher for students without myopia than those with myopia (AOR = 1.263; 95% CI: 1.042–1.530). Students with impairment were 1.321 times more likely to suffer from anxiety than those without it (AOR = 1.321, 95% CI: 1.064–1.641). University students who always consumed high-fat foods had a higher prevalence of anxiety than those who never did (AOR = 1.993, 95% CI: 1.165–3.408, Table 5).

### Factors Associated With Stress Amongst University Students

Following the univariate analysis, factors that had a significant effect on stress were selected for a multifactorial analysis. The results showed that myopia, the presence of impairment, high fatty-food intake, complain of depression and anxiety were significantly associated with complain of stress. Students with no myopia suffered from anxiety 1.3 times more often than those with myopia (AOR = 1.305; 95% CI: 1.022–1.667). Impaired students were more likely to suffer from anxiety than the non-impaired ones (AOR = 1.305, 95% CI: 1.012–1.683). The prevalence of complain of anxiety was higher in university students who always consumed high-fat foods than in those who never did (AOR = 1.285, 95% CI: 1.211–4.312, Table 6).

## Discussion

The DASS-21 questionnaire, although it cannot be considered as a diagnostic tool for psychological disorders, is useful to determine the prevalence of complains of anxiety, depression, and stress.

We found a high prevalence of complains of depression (34.5%), anxiety (33.4%), and stress (12%) in our population. In this survey, a high percentage (62.2%) of the university students had no complains of depression, anxiety, or stress, whilst a low percentage had moderate to severe complains. Compared with other studies (Ramón-Arbués et al., 2020), the present study showed a good overall psychological distress amongst the university students. The commonest mental health problems amongst university students were negative emotional problems (Pedrelli et al., 2015).

Studies of a diverse sample of undergraduate student populations across the globe have also found moderately high rates of mental health problems in this group (Beiter et al., 2015; Bahhawi et al., 2018). The combination of high prevalence of mental health problems and early onset of mental health problems provides particular importance in assessing and addressing mental health problems among undergraduate students. Therefore, focusing on early mental health problems among undergraduate students may also have broad benefits for campus health services and mental health policy development (Beiter et al., 2015).

Studies worldwide have reported differences in the prevalence of psychological distress among university students. Anxiety, depression, and stress are common amongst adolescents and more common in girls than in boys (Wiklund et al., 2012). In the present survey, girls had fewer mental health problems than boys. The author suggests that the higher rate of impairment amongst males than females is one possible reason for the difference in mental health problems between the two. Students who do not live-in halls of residence are more likely to be depressed than those who do. The reason is that living in halls of residence is more connected to peers, thus reflecting the importance of social interaction and peer support in reducing depression (Werner-Seidler et al., 2017). One study also found a significant relationship between myopia and anxiety levels (Li et al., 2020).

In the present survey, complain of anxiety (33.4%) were found to be high in the university student population. The results are consistent with those in previous research conducted amongst general university students. For instance, Blanco et al. (2008) found that the most prevalent mental health problem amongst university students was anxiety.

In addition, this survey showed that students with myopia have higher levels of complain of anxiety and stress, are more likely to sit still for longer periods of time and feel more eyestrain than students without myopia (Li et al., 2018). Meanwhile, the depression levels of students in their fourth year were higher than those in the first year. In addition, senior students are a special population. They are in a critical period of transition between school and society and must make numerous and important decisions that affect their education, lives, and future careers. They may face a variety of negative life events during this critical stage, including changes in their daily lives, problems with relationships, academic difficulties, financial pressures, and the struggle to make important decisions (Zhou et al., 2012). Many issues make senior students more prone to depression than others.

With China’s rapid economic development, the number of children/adolescents with obesity or those who are overweight is increasing and this problem influences their psychological wellbeing (Wang et al., 2019). The high rates of depression and anxiety with high BMI in the present survey suggested that the psychological changes in university students should be considered whilst strengthening interventions for their physical activity. Students with low annual per capita family income were significantly more depressed, family income was positively correlated with individual subjective wellbeing (Hall and Jones, 2007) and the financial stress felt by university students was negatively correlated with their subjective wellbeing (Robb, 2017). Parental education also influenced students’ mental health to some extent, with more educated parents being able to better understand their children’s health behavior and more willing to spend time with them (Lund et al., 2019).

A prospective study found a possible additional dose–response relationship between physical activity and depression (Dunn et al., 2001). The conclusion that physical inactivity is a risk factor for the progression of depression is supported by much evidence from longitudinal observational studies and intervention studies (Pinto Pereira et al., 2014). A significant association was found between depressive symptoms and physical activity and sedentary behavior (Kandola et al., 2020). A review revealed that the beneficial effects of physical activity in patients with depression are compared with those achieved through antidepressants and psychotherapy (Stubbs et al., 2018). Therefore, improving physical activity levels is a priority for maintaining not only physical health but also psychological health.

Regression models suggest that complain of depression, anxiety, and stress are mutual risk factors. Physiological or psychological stress could alter neuroplasticity in the brain whilst increasing the risk of depression and anxiety (Fan et al., 2019). As the three psychological states in the present study are risk factors for one another, psychological complains are possibly confounded with one another and no strict demarcation of boundaries existed; “if you have one symptom, you are likely to have the other as well” (Marshall, 2020).

Physical-activity-related injuries have psychological health effects. In addition, advances in sports medicine have reduced the mean time required for physical resume after PARIs. However, quick physical recovery may not provide ample time for mentality recovery. Meanwhile, poor eating habits, for example, are not necessarily causally related to mental health status. A strong correlation may exist between these covariates and the mental health status amongst Chinese university students. Studies showed that adults with depressive symptoms binge eat more frequently and are less physically active each day than adults without depressive symptoms (Goldschmidt et al., 2014). Symptoms of depression, stress, and anxiety by themselves, without necessarily meeting the full diagnostic threshold for depression, anxiety, and stress disorders, are sufficient to influence health behavioral practices. Individuals with increased psychological symptoms experience more negative emotions and may engage in poor health behaviors in an attempt to reduce distress and/or mitigate the effects of negative emotions (Halperin et al., 2010). All university students, regardless of their psychological state, are likely to be equally susceptible to the effects of their psychological state on health behavior, indicating that maintaining a good psychological state is important for these students.

The innovation of this study is that, firstly, the survey deals with the psychological condition of university students, explores the factors affecting their psychological status including complain of depression, anxiety, stress, paving the way for research related to the mental health of university students.

Secondly, the study had a large sample size and the preliminary literature review provides a good basis for conducting the questionnaire at a later stage. Finally, the method of having student self-reporting is the most practical and cost-effective method at the national level.

However, there are limitations to the study, firstly, there may be recall bias or reporting bias using self-reported questionnaires, particularly in terms of the number of injuries sustained in the past 12 months, and students may not be reporting the most accurate information. Secondly, this study has a cross-sectional design and causal relationships could not be determined. Thus, the diagnosis of psychological symptoms requires other instruments to be formalized. Finally, this study only included university students from rehabilitation-related disciplines nationwide. Future investigations in the areas of depression, anxiety, stress, and other psychological conditions ought to attempt to address these restrictions. Since our research was conducted during COVID-19, we did not consider COVID-19 as a potential risk and analyze its impact on students.

At all events, the findings suggested the necessity to protect and promote the mental health and wellbeing of university students (where applicable).

## Conclusion

In this survey, complain of depression, anxiety and stress were more common; complain of anxiety were more severe than the other two. In some cases, these complains coexist. In addition, the factors associated with these complains were explored. Gender, myopia, impairment, father’s education, fresh vegetables, high-fat foods, and sugar intake were all closely related to psychological distress amongst university students. The findings could contribute to the prophase intervention of psychological disturbances in the university student population, thereby promoting psychological health and wellbeing in this population.

## Data Availability Statement

The original contributions presented in the study are included in the article/supplementary material, further inquiries can be directed to the corresponding authors.

## Ethics Statement

The studies involving human participants were reviewed and approved by the Ethics Committee of The Sixth Hospital of Sun Yat-sen University (IEC Ref: E2020035). The patients/participants provided their written informed consent to participate in this study.

## Author Contributions

YW and YY designed and planned the study. YY acquired and analyzed the data. YY, WY, and JY drafted the manuscript. WY and YX polished and edited the manuscript. YW and DW supervised the whole study. All authors contributed to the concept and design of the study and read and approved the final manuscript for publication.

## Conflict of Interest

The authors declare that the research was conducted in the absence of any commercial or financial relationships that could be construed as a potential conflict of interest.

## Publisher’s Note

All claims expressed in this article are solely those of the authors and do not necessarily represent those of their affiliated organizations, or those of the publisher, the editors and the reviewers. Any product that may be evaluated in this article, or claim that may be made by its manufacturer, is not guaranteed or endorsed by the publisher.
